# Transcatheter Tricuspid Valve Annuloplasty with the Cardioband System: A Step-by-Step Guide

**DOI:** 10.3390/jcm14217772

**Published:** 2025-11-01

**Authors:** Maria Laura Novembre, Lluis Asmarats, Chi Hion Pedro Li, Marcel Santaló-Corcoy, Xavier Millán, Dabit Arzamendi

**Affiliations:** Hospital de la Santa Creu i Sant Pau, Institut de Recerca Sant Pau (IR Sant Pau), Barcelona Sant Antoni M. Claret, 167, 08025 Barcelona, Spain; marialaura.novembre@autonoma.cat (M.L.N.); cli@santpau.cat (C.H.P.L.); msantaloc@santpau.cat (M.S.-C.); xmillan@santpau.cat (X.M.); darzamendi@santpau.cat (D.A.)

**Keywords:** transcatheter, annuloplasty, tricuspid regurgitation

## Abstract

Direct transcatheter tricuspid valve annuloplasty represents a significant advancement in the treatment of severe symptomatic tricuspid regurgitation. Previous studies have shown the efficacy of transcatheter annuloplasty with significant reductions in tricuspid regurgitation and improvements in functional status. The aim of this review is to provide a comprehensive step-by-step guide to the procedure, focusing on appropriate patient selection, main procedural steps and identification and management of possible complications to enhance our understanding of the procedure and maximize procedural success.

## 1. Introduction

Tricuspid regurgitation (TR) is a common disease, occurring in any degree in 70% to 90% of the general population [1]. Severe TR affects nearly 4% of the population over 75 years, has a higher incidence in women and in the elderly population, and carries a grim prognosis irrespective of ventricular function, pulmonary artery pressure or other comorbidities [1].

TR remains largely undertreated due to high in-hospital mortality rates associated with tricuspid valve surgery (~10%) [2]. In the last two decades, significant advancements have been made in the field of transcatheter therapies for treating TR, including leaflet approximation therapies (tricuspid edge-to-edge repair), transcatheter annuloplasty devices, orthotopic tricuspid valve replacement and heterotopic tricuspid valve replacement [1,3]. Whereas tricuspid edge-to-edge repair has been the most widely used therapy so far, a non-negligible proportion of patients have been considered unsuitable for tricuspid edge-to-edge repair because of relative anatomical contraindications (e.g., leaflet thickening, large coaptation gaps, multileaflets anatomies with multiple jets) [4].

An in-depth assessment of the mechanism and etiology of TR is paramount to determine the most appropriate device for every patient. TR can be divided into primary or organic due to anatomical abnormalities of the valve (<10%) and secondary or functional TR caused by dilatation of the tricuspid annulus due to right cardiac chamber enlargement, such as right ventricular dilation in pulmonary hypertension or right atrial enlargement in atrial fibrillation, which accounts for up to 90% of the cases. More recently [5], a novel classification regarding the pathophysiological characterization of TR has been proposed: primary TR (due to intrinsic abnormalities of the tricuspid valve), atrial TR (due to dilatation of the right atrium and tricuspid annulus secondary to atrial fibrillation), ventricular TR (secondary to dilation and/or dysfunction of the right ventricle in patients with pulmonary hypertension and right ventricular pathologies) and secondary to implantable devices [1].

The pathophysiology of atrial tricuspid regurgitation (TR) is characterised by atrial dilatation, typically resulting from chronic pressure or volume overload secondary to long-standing atrial fibrillation or left-sided heart failure. This leads to annular dilatation and remodelling, particularly along the anterior and posterior portions, which flattens the normal saddle shape of the annulus and disrupts leaflet coaptation. The resulting malcoaptation increases haemodynamic stress, further promoting atrial dilatation and creating a self-perpetuating cycle of progressive regurgitation.

Surgical annuloplasty remains a common option of choice for treating functional TR and involves placing a prosthetic ring or band to reshape and reduce the size of the tricuspid annulus, improving leaflet coaptation and restoring valve function.

In recent years, transcatheter tricuspid annuloplasty has emerged as an alternative therapy for severe functional TR in patients deemed at high risk for surgery [6]. The Cardioband system (Edwards Lifesciences, Irvine, CA, USA) is a transcatheter, adjustable annuloplasty device designed to simulate the effects of surgical annuloplasty without requiring open-heart surgery. It replicates the physiological benefits of surgical techniques by reducing the dimensions of the tricuspid annulus, addressing the primary mechanism of functional TR [7].

## 2. Device Characteristics

**Figure 1:**
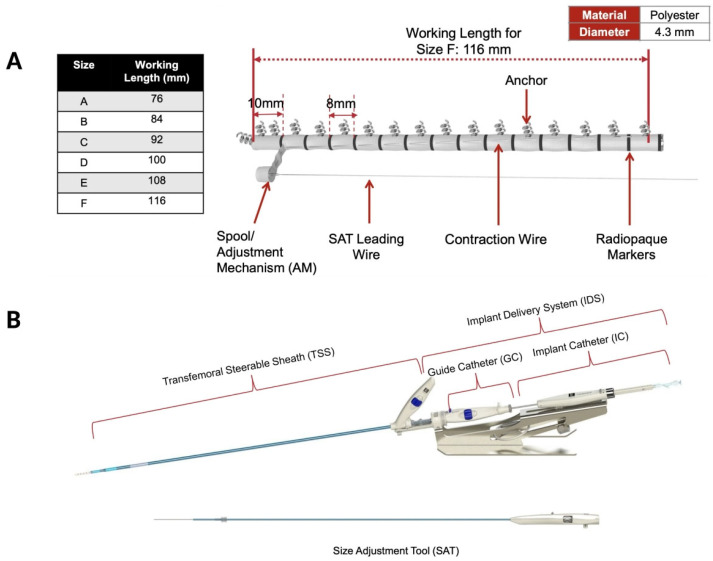
Components of the Cardioband Tricuspid Valve Repair System. (**A**) Cardioband implant. (**B**) Implant delivery system including a steerable guiding catheter and an implant catheter. (Courtesy of Edwards Lifesciences).

The Cardioband transcatheter tricuspid annuloplasty system is inserted via transfemoral venous access using a 26F delivery system [8]. The device received the CE-mark approval in 2015 for mitral annuloplasty and in 2018 for tricuspid annuloplasty [9]. The Cardioband system consists of a polyester band incorporating a contraction mechanism and radiopaque markers for visualisation, a hydrophilic release system for implant introduction, deployment and adjustment, and anchoring motors that deliver up to 17 stainless steel anchors to secure the device to the tricuspid annulus (Figure 1A). Each anchor measures 6 mm in length and 2.6 mm in diameter and has a spiral, screw-type design to ensure firm attachment to the annular tissue. The device is available in six sizes (A–F), with working implant lengths ranging from 76 to 116 mm, covering tricuspid annular dimensions (from the aorta to the coronary sinus) between 73 and 120 mm.

The Cardioband delivery system includes both the implant delivery system and a steerable sheath (TSS) (Figure 1B). The implant delivery system comprises a steerable guiding catheter and an implant catheter (IC) with the implant device mounted at its distal tip. Finally, the size adjustment tool (SAT) connects to the implant wire spool and controls band contraction, allowing up to a 30% reduction in the annular perimeter during cinching.

## 3. Pre-Procedural Anatomical Screening

### 3.1. Transthoracic/Transesophageal Echocardiography

Transthoracic and transesophageal echocardiography (TEE) are the cornerstone imaging modalities for assessing the severity and mechanism of TR, tricuspid annular size, number of leaflets and morphology, right ventricular size and function, and determine whether the acoustic window is sufficient to ensure high-quality guidance during transcatheter procedures.

Device selection should be based on the degree of annular dilatation, jet location and coaptation gap size [1]. Patients with annular dilatation and mild or moderate tethering are in general good candidates for both edge-to-edge and annuloplasty repair therapies. The former may be preferred for commissural jets with reasonable coaptation gaps (8.5 mm), whereas annuloplasty may be prioritized for treating central jets with annular dilatation as the primary mechanism of TR.

From an echocardiographic standpoint, the ideal candidate for percutaneous tricuspid annuloplasty should have atrial functional TR, central jet location, with no severe tethering (tethering height <1.0 cm, tenting area <2.5 cm^2^ and 3D-tenting volume <3.5 mL) and sufficient landing zone for device anchoring (Table 1) [4].

In patients with pre-existing cardiac implantable electronic devices (CIEDs), the lead position and its interaction with the leaflets should be carefully evaluated, particularly in the transgastric view. Pacemaker leads that do not impinge on the leaflets are compatible with transcatheter annuloplasty. However, leaflet impingement by a CIED lead, organic disease (prolapse, flail, carcinoid, or congenital abnormalities), leaflet restriction or perforation, excessive annular dilatation (beyond device range), or prior surgical annuloplasty may represent unfavourable anatomies for this therapy.

As with other transcatheter tricuspid interventions, patients should ideally exhibit preserved left ventricular function, normal or mildly reduced right ventricular function (TAPSE >13 mm), no pre-capillary pulmonary hypertension (confirmed by invasive right heart catheterisation), and no more than mild hepatic or renal dysfunction.

### 3.2. Cardiac Computed Tomography

Cardiac computed tomography (CT) is the imaging modality of choice for the preprocedural assessment, allowing accurate measurement of the tricuspid annulus, exclusion of annular calcification, evaluation of tissue quality and right coronary artery (RCA) proximity, prediction of optimal fluoroscopic views and, ultimately, selection of the appropriate band size for each patient.

CT imaging of the tricuspid annulus should be performed during the mid- to late-diastolic phase of the cardiac cycle, typically between 60% and 90%. In clinical practice, retrospective ECG-gated acquisition is often preferred, as it captures images throughout the cardiac cycle, enabling the selection of diastolic frames. These can then be reconstructed in both two-dimensional (axial, sagittal, and coronal) and three-dimensional volumes, which are essential to visualise the full shape of the tricuspid annulus, often asymmetrical or elliptical.

**Figure 2:**
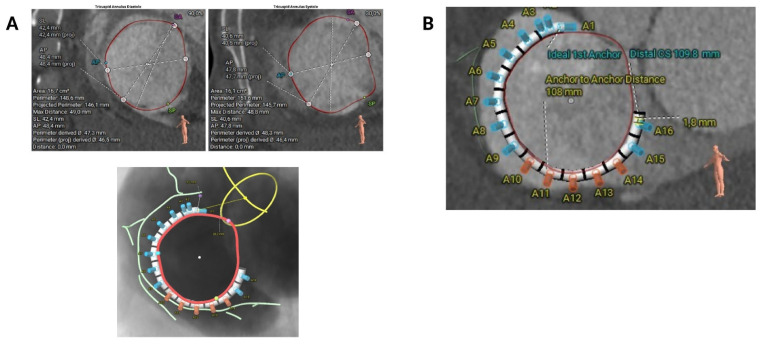
Key measurements for Cardioband sizing selection. (**A**) Main dimensions of the tricuspid annulus, including anteroposterior diameter, septolateral diameter and annular perimeter. (**B**) Tricuspid valve annulus measurement from the aorta (first intended anchor) to the distal coronary sinus to determine the appropriate band size.

Key measurements of the tricuspid annulus include the anteroposterior diameter, septolateral diameter, and annular perimeter (Figure 2A). It is essential to measure the annulus at the first optimal point as close as possible to the aorta (usually 2–3 cm from the centre of the aortic valve), where the RCA ostium disappears and adequate tissue is present towards the distal coronary sinus. This step is crucial for selecting the correct implant size (Figure 2B). Table 2 provides the recommended band sizes according to the tricuspid annular hemiperimeter (sizing chart).

**Figure 3:**
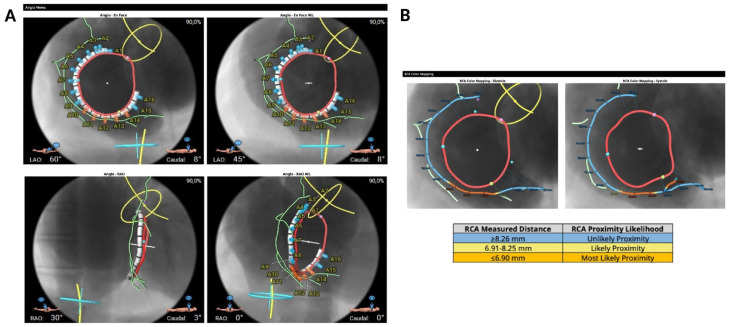
Computed tomography-based evaluation for tricuspid annuloplasty procedural planning. (**A**) Computed tomography-based fluoroscopic working views (face and right anterior oblique) (**B**) Proximity between the tricuspid annulus and the right coronary artery.

It is possible to reconstruct the valvular annulus and predict the most suitable fluoroscopic projections for implantation (Figure 3A). Anatomical suitability for transcatheter annuloplasty includes an ideal safety margin between the annulus and the RCA of 6.9 mm (with a minimal required distance of 2.6 mm, equivalent to the anchor diameter) (Figure 3B) a right atrial height of less than 90 mm, a distance from the first anchor to the distal coronary sinus of less than 120 mm (Table 3).

Finally, assessment of the inferior vena cava (IVC) ostium is also important during screening, as it provides valuable information on the catheter trajectory and entry into the right atrium.

## 4. Procedural Steps

The procedure is conducted under general anesthesia and TEE guidance, through right venous femoral access (26F). In addition, an arterial access (6F) is needed to cannulate the RCA and advance a radiopaque guidewire for fluoroscopic reference.

### 4.1. TSS and IC Positioning in the Right Atrium

The TSS is advanced over a super-stiff guidewire into the right atrium. The implant delivery system is then inserted through the TSS, and the tip of the IC is oriented toward the anteroseptal commissure.

### 4.2. IC Navigation

Integration of echocardiographic and angiographic guidance is essential to ensure accurate and safe navigation of the device. The mid-esophageal bicaval view plays a pivotal role at this stage, helping to monitor the TSS and guide catheter as they traverse the right atrium, confirming correct orientation and the mid-esophageal short-axis view may help assessing the distance to the aorta [10,11].

**Figure 4:**
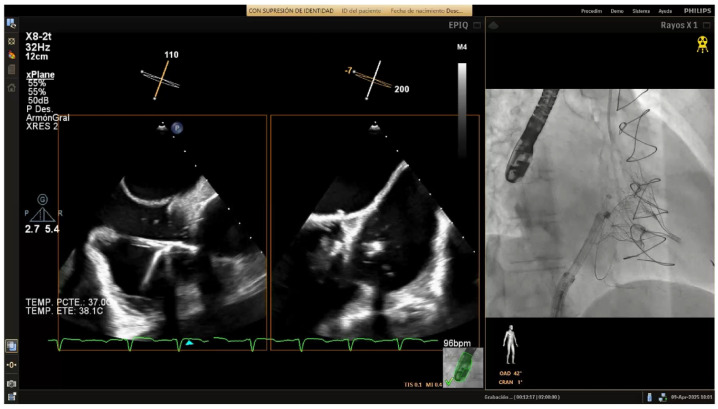
Delivery system navigation. The mid-esophageal bicaval view supports real-time visualization of the steerable sheath and guide catheter, while the mid-esophageal short-axis view aids in assessing the distance to the aorta. As the guide catheter nears the tricuspid valve, fluoroscopic left anterior oblique projection helps identifying the anteroseptal commissure and avoiding the right coronary artery.

As the guide catheter approaches the tricuspid valve (Figure 4), it is important to avoid structural interference or leaflet damage. Angiographically, the left anterior oblique projection helps visualize the anteroseptal commissure of the tricuspid annulus and ensures avoidance of the RCA course. Echocardiographically, transgastric views help visualize the anterior and posterior annulus, while high, mid, and deep esophageal views are useful for assessing the lateral annulus.

After the catheter is near the anteroseptal commissure, TEE and angiographic views are used together to check the IC tip alignment as it moves towards the tricuspid annulus. RAO projections allow visualization of the IC’s approach to the annulus with respect to its surrounding anatomy in order to confirm that the catheter’s tip follows the intended trajectory towards the target anchor points and is not obstructed by the leaflet or at risk of inducing injury. TEE bi-plane views are then employed to monitor precise positioning in relation to the hinge points and to ensure that the IC makes accurate contact with the annular tissue.

Any deviation from the hinge point region can compromise device efficacy, so continuous checks are essential to avoid positioning errors. If the IC alignment appears compromised at any stage, TEE enables quick reassessment to confirm or correct catheter orientation relative to the tricuspid valve.

### 4.3. Annuloplasty Device Implantation (Anchoring)

The anchoring phase is critical for the secure placement of the Cardioband around the tricuspid annulus. It is performed in a clockwise sequence, starting from the anteroseptal commissure near the aorta.

#### 4.3.1. First Anchor Positioning (Anteroseptal Commissure)

**Figure 5:**
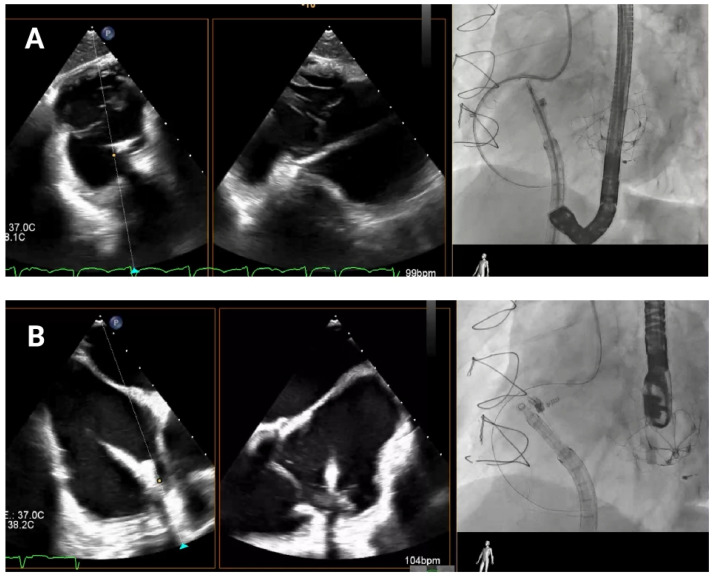
Anterior tricuspid annulus anchoring. (**A**) First anchor placement in the fibrous tissue anterior to the tricuspid annulus, near the aorta. Orientation is confirmed using mid-esophageal right ventricular inflow-outflow and transgastric right ventricular basal views, along with fluoroscopy in an RAO projection. (**B**) Subsequent advancement of the implant catheter along the anterior annulus through transgastric and mid-esophageal views (40–70°) and multiplanar reconstruction.

The first anchor is positioned in the fibrous tissue anterior to the tricuspid annulus, close to the aorta, at least 25 mm from the center of aortic valve (Figure 5A). The mid-esophageal right ventricular inflow-outflow view and transgastric right ventricular basal views are used to confirm the orientation of the IC [10,11]. It is essential that the IC tip is directed away from the aortic root to avoid complications. Fluoroscopy is performed using the LAO and RAO projections to verify directional accuracy, ensuring that the catheter does not misalign toward the aortic root or the RCA.

#### 4.3.2. Anterior Annulus Anchoring

Once the first anchor is released, the procedure progresses toward the anterior annulus (Figure 5B). TEE views, such as the transgastric views at 40–60° and mid-esophageal views at 50–70°, provide crucial information for visualizing the IC’s position along the anterior segment of the annulus [10,11]. Biplane and multiplanar reconstruction techniques enhance visualization, allowing for more accurate device placement. Fluoroscopically, the RAO projection guides the implant catheter (IC) along the annulus and ensures correct anchor positioning relative to the valvular structures. Before release, a “push–pull” test is performed under combined echocardiographic and fluoroscopic (RAO) guidance. On echocardiography, pulling allows observation of the annulus being drawn toward the IC (Supplemental Video S1). On fluoroscopy, pulling causes the anchor to move slightly from its original position, resulting in traction of the previously deployed band, anchors, and the RCA wire.

The “push-pull test” is considered satisfactory if:

- A ≥2 mm annular displacement is observed;
- Synchronous movement of anchor–catheter–guidewire occurs;
- No leaflet traction is detected.

Once anchoring is confirmed to be effective and stable, the anchor is released.

#### 4.3.3. Anteroposterior Commissure

As the IC approaches the anteroposterior commissure, visualization becomes more complex due to angulation. High-resolution transgastric views and upper esophageal perspectives at 60–90° are used for clarity. The transgastric 150° view can yield excellent resolution while an upper esophageal 3D view with multiplanar reconstruction mitigates device shadowing, allowing visualization of the annulus [10,11], Fluoroscopic imaging is essential here to ensure that the anchor is being positioned correctly without obstruction or misalignment.

#### 4.3.4. Posterior Annulus (Hooking)

**Figure 6:**
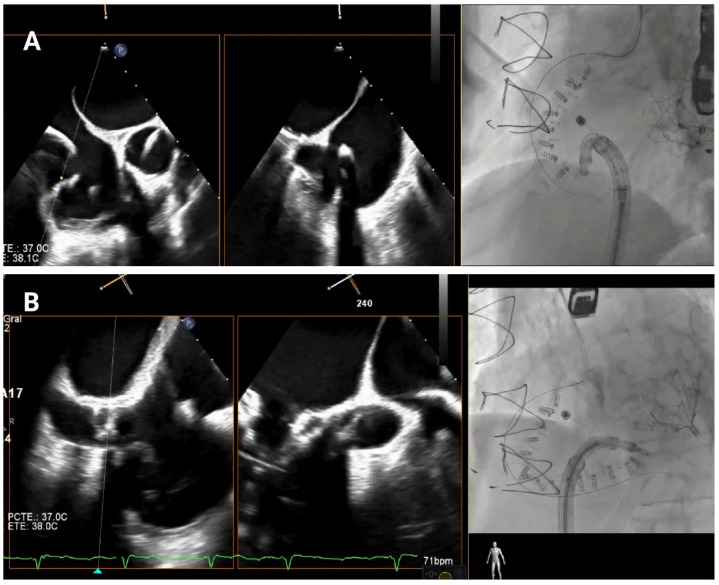
Posterior tricuspid annulus anchoring. (**A**) Posterior annulus anchoring requires navigating anatomical complexity due to echo beam alignment and distance from the probe. A 90° “hooking” angle between the implant catheter and the transvenous steerable sheath is often necessary to overcome the right coronary artery and access the posterior annular region. Transgastric transesophageal view (150°) and upper esophageal retroflexed views aid visualization, while RAO and LAO fluoroscopic projections confirm alignment and proper catheter trajectory. (**B**) Final anchoring at the posteroseptal commissure using deep and mid-esophageal bicaval views (0–135°) for visualization of the coronary sinus and septum. RAO projection and biplane imaging help verifying proper anchor placement and avoiding overlap or misalignment with nearby structures.

Anchoring along the posterior annulus (Figure 6A) poses unique challenges due to its parallel alignment with the echo beam and distance from the probe. At this point, the device often needs to be curved in an angle of 90° between the IC and the TSS (“hooking”) to cross the RCA and properly reach the anchor points (Supplemental Video S2). A mid-esophageal MPR view and a transgastric view at 150° provide a better view of this segment. RAO and LAO projections are employed to verify the alignment of the IC with the annulus and ensure there is no contact with unintended structures [10,11]. If loss of coaxiality or echo shadowing occurs, corrective actions include sheath repositioning, modification of LAO/RAO angles, or pre-shaping of the catheter curve.

#### 4.3.5. Posteroseptal Commissure

Finally, as the band approaches the posteroseptal commissure (Figure 6B), deep esophageal and mid-esophageal bicaval views ranging from 0° to 135° may facilitate visualization of the coronary sinus and the septum, which are critical during this phase [10,11]. For final anchors placement, the RAO view helps confirming anchor security and alignment with the valve annulus, while avoiding any potential misalignment or overlap with other structures. Biplane imaging enhances alignment checks and reduces shadowing artifacts.

### 4.4. Band Cinching

**Figure 7:**
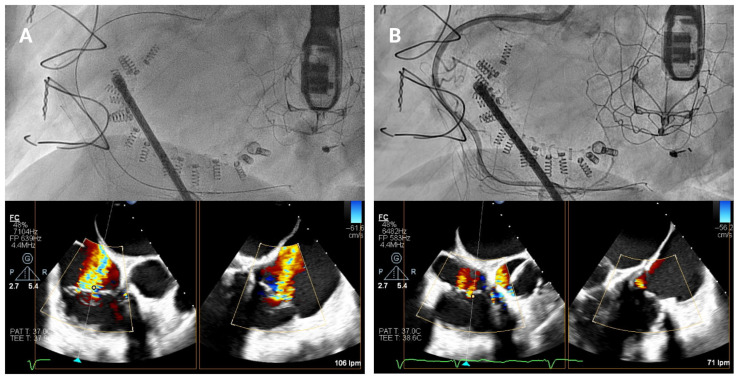
Band cinching and post-implant assessment. The size adjustment tool is used to cinch the Cardioband, reducing the tricuspid annular diameter by 3.5–5.5 cm depending on the band size. Pre-cinching (**A**) and final result post-cinching (**B**).

In the final release phase of the procedure, the size adjustment tool (SAT) is advanced to contract the Cardioband implant (Figure 7), a crucial step in securing the device around the tricuspid annulus and effectively reducing the diameter. This reduction is performed in incremental stepwise adjustments, which range between 0 cm and 2.5–3.5–4.5–5.5 cm depending on the chosen band size, to achieve optimal tension and alignment with the annulus. Continuous real-time monitoring with TEE and fluoroscopic imaging is essential during this phase to confirm successful positioning and ensure gradual tightening without impinging on surrounding structures or compromising valve function.

### 4.5. Final Result Assessment

Post-implantation, comprehensive imaging is used to assess final TR reduction, final transvalvular gradient and assess right ventricular function. Coronary angiography specifically assesses the RCA for any bending or impingement.

## 5. Complications and Troubleshooting

Potential complications during the Cardioband procedure include RCA injury, tricuspid leaflet injury, conduction disturbances, pericardial effusion or thrombus formation.

RCA deformation is generally reversible and benign. Gerçek et al. [12]. analyzed 14 patients who developed acute RCA deformation during a Cardioband procedure. In most of these patients (12/14, 80%), the deformation was severe (>80% angiographic stenosis) but did not result in significant flow impairment, vessel damage, or myocardial infarction. Creatine kinase levels showed no evidence of myocardial injury. Follow-up coronary angiography demonstrated complete reversal of right coronary artery (RCA) deformation in all patients after a mean of 5.36 days. Two patients underwent RCA stenting without evidence of true vascular injury.

**Figure 8:**
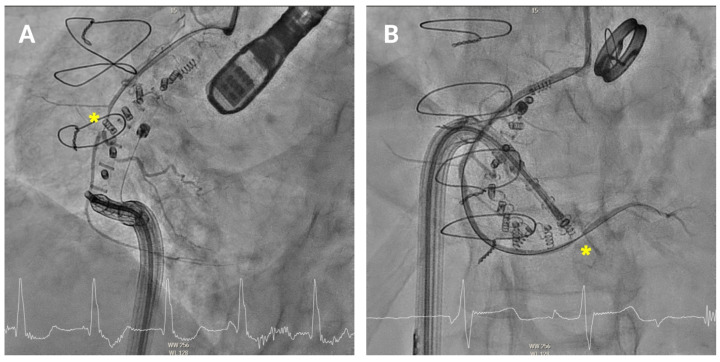
Pinching of the right coronary artery. Coronary angiography showing anchor-related pinching (asterisk) of the mid (**A**) and distal (**B**) right coronary artery.

RCA pinching may rarely occur due to mechanical compression of the artery, either from closely positioned anchors or excessive tightening of the Cardioband (Figure 8). This compression can restrict coronary blood flow, potentially leading to myocardial ischaemia. Patients with RCA pinching may present with chest pain, dyspnoea, or ECG changes suggestive of ischaemia. When RCA pinching is suspected, immediate coronary angiography should be performed to confirm the compression and allow for repositioning of the affected anchor. In most cases, the pinching resolves after anchor repositioning and intracoronary nitroglycerin administration. In the rare event of persistent, flow-limiting coronary compression or confirmed extrinsic adventitial injury on intracoronary imaging, urgent intervention—such as balloon angioplasty or stent implantation—may be necessary to restore normal coronary perfusion.

Moreover, it is essential to address other potential procedural complications, particularly vascular and bleeding events that may occur during venous access, device introduction, or anchor deployment. These can include access-site haematomas, vessel perforation, and pericardial effusion resulting from right atrial injury, with subsequent major bleeding that may require transfusion or surgical repair. Although uncommon, new-onset conduction disturbances may develop during transcatheter tricuspid annuloplasty due to the proximity of the tricuspid valve to the atrioventricular node and the His bundle. Finally, improper anchor placement may lead to tricuspid leaflet injury with residual tricuspid regurgitation, or even cardiac perforation, given the close relationship between the tricuspid valve, right atrial wall, and inferior vena cava. In addition to acute events, late complications such as Cardioband device dislocation have been described and may necessitate surgical intervention or transcatheter reintervention. A published case series has reported delayed device-related failures following mitral transcatheter annuloplasty, underscoring the importance of structured long-term follow-up [13].

## 6. Peri-Procedural Medication Management

Anticoagulation during the procedure should be maintained with an activated clotting time (ACT) between 250 and 300 s, and heparin reversal with protamine should be reserved for cases of major bleeding only. From our experience, following the procedure, patients in sinus rhythm are generally treated with single antithrombotic therapy. In patients with atrial fibrillation, which represents the majority of patients, continuation of oral anticoagulation is mandatory. Prophylaxis against infective endocarditis is recommended, typically consisting of a single dose of a first-generation cephalosporin administered before vascular access.

## 7. Follow-Up and Efficacy Assessment

Regular clinical follow-up is essential to assess device stability, detect potential recurrence of tricuspid regurgitation, and monitor right heart remodelling over time. From our experience, a structured schedule is recommended, including transthoracic echocardiography and electrocardiography at discharge, followed by a clinical evaluation and transthoracic echocardiography at 90 days. Comprehensive reassessments should then be performed at 12 months combining imaging and functional testing. During each follow-up visit, several key parameters should be systematically evaluated including the severity of tricuspid regurgitation, the percentage reduction in annular perimeter, the transvalvular mean gradient and right ventricular function. Clinical and functional outcomes should also be monitored, including New York Heart Association functional class, six-minute walk test distance, and the Kansas City Cardiomyopathy Questionnaire score. The long-term objectives of follow-up are the maintenance of tricuspid regurgitation at or below a moderate grade, improvement in right ventricular function, and sustained symptomatic benefit at one year or longer.

## 8. Evidence and Outcomes of Cardioband for Tricuspid Regurgitation

Cardioband transcatheter annuloplasty has emerged as an effective therapy for patients with severe or torrential functional TR who are at high surgical risk. A single-center study by Pardo Sanz et al. [14]. demonstrated that Cardioband implantation achieved a significant and sustained reduction in septolateral annular diameter (−10.4 mm, *p* < 0.001) and TR severity, with 82% of patients improving to NYHA functional class I–II and showing marked reduction in peripheral edema at one year. A meta-analysis including 991 patients who underwent isolated transcatheter tricuspid valve repair showed that annuloplasty-based systems (including Cardioband) significantly reduced ≥ severe TR and NYHA class III–IV, albeit with slightly higher bleeding rates compared to edge-to-edge repair [15]. Similarly, a systematic review and meta-analysis by Piragine et al. [16], which included 11 Cardioband studies (≈200 patients), showed consistent annular diameter reduction (−9.31 mm), vena contracta decrease (−6.41 mm), and EROA improvement (−0.50 cm^2^). TR reduction occurred in 91% of patients, functional improvement in 63%, and 24-month survival and freedom from heart failure hospitalization were 80% and 58%, respectively. Overall, Cardioband demonstrates durable efficacy in annular remodeling and symptom relief with an acceptable procedural safety profile, supporting its role as a feasible non-surgical option for functional TR in high-risk patients. More recently, in a study involving 58 patients with severe functional TR, Cardioband reduced TR by ≥2 grades in 83% of patients and significantly improved right-heart hemodynamics—increasing cardiac index (from 2.4 to 2.8 L/min/m^2^), pulmonary artery pulsatility index (from 1.73 to 2.13), and right-ventricular power index (from 0.15 to 0.21 W/m^2^), while lowering right-atrial pressure and x-v descent height (from 12.5 to 7 mmHg, *p* < 0.001) [17]. Post-procedural x-v descent height emerged as an independent predictor of 2-year survival (*p* = 0.002), correlating with sustained TR reduction and right-ventricular reverse remodeling. These findings confirm Cardioband’s efficacy in achieving anatomical and hemodynamic restoration, translating into improved function and survival with an acceptable safety profile in advanced TR (Table 4).

## 9. Cardioband Among TEER and TTVR

The Cardioband system represents a major advancement in the percutaneous treatment of functional tricuspid regurgitation. Its design mimics surgical annuloplasty by directly reducing annular dimensions and restoring leaflet coaptation without the need for open-heart surgery. Clinical studies have consistently demonstrated significant reductions in TR severity, improvement in NYHA functional class, and increased exercise tolerance following Cardioband implantation [14,15,16,17]. However, procedural complexity and anatomical limitations still restrict its broader application. Compared with edge-to-edge repair systems, annuloplasty provides a more physiological correction of annular dilatation but requires favourable annular anatomy and adequate tissue quality. Conversely, orthotopic valve replacement ensures complete TR elimination but is associated with higher procedural risk and potential complications such as conduction abnormalities or thrombosis. The choice among these modalities depends on anatomical feasibility, patient comorbidities, and operator expertise.

## 10. Conclusions

In conclusion, transcatheter tricuspid annuloplasty represents an appealing alternative for high-risk surgical patients with severe symptomatic tricuspid regurgitation, particularly those with atrial TR who are unsuitable for edge-to-edge repair. Comprehensive multimodality imaging—including TEE, CT, and angiography—is essential to ensure optimal outcomes and minimise complications. However, the emergence of orthotopic prostheses—some of which are already commercially available—featuring a more standardized and straightforward implantation process, could compete directly with annuloplasty techniques and, depending on comparative results and clinical adoption, might lead to the strategic deprioritization or even discontinuation of Cardioband. As the percutaneous tricuspid valve treatment toolbox continues to expand, transcatheter annuloplasty may remain a valuable option, particularly for patients with atrial TR.

## Figures and Tables

**Table 1:** Favorable anatomic characteristics for Cardioband.

Characteristic	Ideal Requirement
Type of TR	Atrial functional TR
Central Jet Location	Central location
Severity of Tethering	Not severe
Tethering Height	<1.0 cm
Tenting Area	<2.5 cm^2^
3D Tenting Volume	<3.5 mL
Landing Zone	Sufficient for device anchoring

TR: tricuspid regurgitation.

**Table 2:** Computed tomography-based sizing chart.

Deployment Length (mm)	Implant Size	SAT Max Cinching (cm)	Anchors Required
73–80	A	3.5	12
81–88	B	4	13
89–96	C	4.5	14
97–104	D	5	15
105–112	E	5.5	16
113–120	F	5.5	17

SAT: Size Adjustment Tool.

**Table 3:** Relationship with surrounding structures.

Criteria	Ideal Requirements	Intermediate Cautious Zone	Unfavorable
RCA patency	Patent	-	Chronic total occlusion
Distance from the TA to the RCA	≥6.9 mm	2.6–6.9 mm	<2.6 mm (first 3 anchors or ≥6 consecutive anchors)
Right atrium	50–70 mm	71–90 mm	≥90 mm
Distance from the first anchor to distal CS	100–120 mm	121–139	>139 mm

CS: coronary sinus; RCA: right coronary artery; TA: tricuspid annulus.

**Table 4:** Evidence and Outcomes of Cardioband for Tricuspid Regurgitation.

Study (Year)	Design/Population	Follow-Up	Efficacy Outcomes	Safety Outcomes
Pardo Sanz et al. [14], EHJ Imaging 2022	Prospective single-center, n = 24 (≥severe functional TR)	Mean 9 mo (1 yr subset)	Annular diameter −10.4 mm (*p* < 0.001); TR grade reduced; 81.8% NYHA I–II; 6MWD +69 m	Technical success 91.6%; 1 death; no major complications
Alperi et al. [15], Rev Esp Cardiol 2023	Meta-analysis of 19 TTVr studies (991 pts; annuloplasty and edge-to-edge)	Early + mid-term (7.8 mo mean)	Severe TR RR 0.33 (95% CI 0.26–0.42); Vena contracta −5.9 mm; RV EDD −3.7 mm; NYHA III–IV RR 0.32	30-day mortality 2.8%; stroke 0.2%; bleeding 13% (annuloplasty vs. 3.8% edge-to-edge)
Piragine et al. [16], J Clin Med 2024	Systematic review + meta-analysis (11 studies; ≈200 pts)	Up to 24 mo	Δ annular diam −9.31 mm [95% CI −11.47; −7.15]; Δ vena contracta −6.41 mm; Δ EROA −0.50 cm^2^; TR improvement 91%; NYHA improvement 63%	24-mo survival 80.1%; freedom from HF hosp. 57.8%; low procedural mortality
Althoff et al. [17] Circ Cardiovasc Interv 2025	Retrospective multicentre (58 pts with severe-torrential functional TR)	2 ys	TR ↓ ≥2 grades in 83%; Cardiac index 2.4 → 2.8 L/min/m^2^; PAPi 1.73 → 2.13; RV power 0.15 → 0.21 W/m^2^; XV height 12.5 → 7 mm Hg (*p* < 0.001)	High procedural success rate; acceptable complication rate; low mortality. Lower post-TTVA XV height independently predicted 2-year survival (*p* = 0.002) and RV remodeling (*p* < 0.001). Cardioband improves hemodynamics and prognosis.

## Data Availability

No new data were created or analyzed in this study. Data sharing is not applicable to this article.

## Supplementary Materials

The following supporting information can be downloaded at: https://www.mdpi.com/article/10.3390/jcm14217772/s1, Video S1. Illustration of a “push-pull” test under echocardiographic guidance. Video S2. Illustration of a hooking maneuver (90° angle between the IC and the TSS before the 9th anchor) under fluoroscopic guidance.

## Abbreviations

The following abbreviations are used in this manuscript:

CIED	Cardiac implantable electronic devices
CT	Computed tomography
IC	Implant catheter
RAO	Right anterior oblique
RCA	Right coronary artery
TEE	Transesophageal echocardiography
TR	Tricuspid regurgitation
TSS	Transfemoral Steerable Sheath
